# Characteristics of bibliometric analyses of the complementary, alternative, and integrative medicine literature: A scoping review protocol

**DOI:** 10.12688/f1000research.130326.1

**Published:** 2023-02-13

**Authors:** Jeremy Y. Ng, Henry Liu, Aimun Qadeer Shah, L. Susan Wieland, David Moher

**Affiliations:** 1 Department of Health Research Methods, Evidence, and Impact, Faculty of Health Sciences, McMaster University, Hamilton, Ontario, Canada; 2 Centre for Journalology, Clinical Epidemiology Program, Ottawa Hospital Research Institute, Ottawa, Ontario, Canada; 3 Center for Integrative Medicine, University of Maryland School of Medicine, Baltimore, Maryland, USA; 4 School of Epidemiology and Public Health, Faculty of Medicine, University of Ottawa, Ottawa, Ontario, Canada

**Keywords:** bibliometric analysis, complementary and alternative medicine, integrative medicine, scientometric analysis, scoping review

## Abstract

**Background:** There is a growing body of literature on complementary, alternative, and integrative medicine (CAIM), which offers a holistic approach to health and the maintenance of social and cultural values. Bibliometric analyses are an increasingly commonly used method employing quantitative statistical techniques to understand trends in a particular scientific field. The objective of this scoping review is to investigate the quantity and characteristics of evidence in relation to bibliometric analyses of CAIM literature.

**Methods:** The following bibliographic databases will be searched: MEDLINE, EMBASE, PsycINFO, AMED, CINAHL, Scopus and Web of Science. Studies published in English, conducting any type of bibliometric analysis involving any CAIM therapies, as detailed by an operational definition of CAIM adopted by Cochrane Complementary Medicine, will be included. Conference abstracts and study protocols will be excluded. The following variables will be extracted from included studies: title, author, year, country, study objective, type of CAIM, health condition targeted, databases searched in the bibliometric analysis, the type of bibliometric variables assessed, how bibliometric information was reported, main findings, conclusions, and limitations. Findings will be summarized narratively, as well as in tabular and graphical format.

**Conclusions:** To the best of our knowledge, this scoping review will be the first to investigate the characteristics of evidence in relation to bibliometric analyses on CAIM literature. The findings of this review may be useful to identify variations in the objectives, methods, and results of bibliometric analyses of CAIM research literature.

## Introduction

Complementary, alternative, and integrative medicine (CAIM) is a complex term referring to three distinct concepts related to the use of non-conventional medicine. ^1^^,^^2^ “Complementary medicine” describes non-conventional therapeutic approaches that are used together with conventional therapies. ^1^ “Alternative medicine” describes non-conventional therapeutic approaches used in replacement of conventional therapies. ^1^ “Integrative medicine” describes the combined use of both conventional and non-conventional therapies in a coordinated manner. ^1^^,^^2^ For the purpose of this study, each of these approaches may also incorporate elements of “traditional medicine” which is the “knowledge, skills and practices based on the theories, beliefs and experiences indigenous to different cultures, used in the maintenance of health and in the prevention, diagnosis, improvement or treatment of physical and mental illness”. ^3^ All these concepts will be collectively referred to as “complementary, alternative, and integrative medicine” (abbreviated as CAIM).

CAIM practitioners often emphasize a holistic approach to health, including the consideration of cultural and social values. ^2^^,^^4^ Clients often perceive CAIM as better at providing individualized, person-centred care, compared to mainstream health approaches. ^5^^,^^6^ Prevalence of CAIM use is increasing worldwide, and, accordingly, the body of literature on CAIM research has grown immensely, with the steepest increase in CAIM publications observed between the mid-2000s and mid-2010s. ^7^^,^^8^ It is of interest to determine broad research trends of CAIM research literature, and identify specific CAIM topics explored (e.g., acupuncture, aromatherapy). While some CAIM therapies (e.g., yoga for depressive symptoms, ^9^ exercise therapy for reducing falls in older people ^10^) have been shown to be safe and effective, many other therapies have insufficient evidence to demonstrate their effectiveness or safety. ^11^^,^^12^ Furthermore, even when basic effectiveness and safety are established, questions often remain about key characteristics such as intervention dose and implementation or applicability to different patient populations and settings. Bibliometric analyses can be used to detect knowledge gaps and to identify research trends that help predict whether such knowledge gaps are likely to be met.

Bibliometric analysis involves the application of quantitative statistical techniques to bibliometric data (e.g., total number of citations, total number of publications) and can be used for a variety of purposes, such as identifying patterns in a given field of research. ^13^ Bibliometric analysis techniques can broadly belong to categories of performance analysis (i.e., techniques measuring contributions of research constituents) or science mapping (i.e., techniques measuring relationships between research constituents). ^13^ Examples of research constituents include authors, countries, institutions, and topics. ^13^ Performance analysis techniques can further be divided into publication-related metrics (e.g., total number of publications), citation-related metrics (e.g., average citations, total number of citations), and citation-and-publication-related metrics (e.g., h-index, g-index, proportion of cited publications). ^13^ Science mapping techniques can include methods such as citation analysis, co-citation analysis, bibliographic coupling, co-word analysis, and co-authorship analysis. ^13^ For instance, co-citation analysis examines the frequency of publications being cited together, which may reveal thematic clusters. ^13^ Enrichment techniques of network metrics (i.e., quantitative measures of research constituents’ relative importance), clustering (i.e., grouping of similar objects using clustering algorithms), and visualization (i.e., graphical visualizations of research constituents’ connections) can be employed to enhance understanding of science mapping techniques. ^13^ For instance, software like VOSviewer can be used to graphically visualize thematic clusters in co-citation analysis. ^13^

Advantages of bibliometric analyses include facilitating the examination of large datasets that are not feasible for investigation by manual review (e.g., literature reviews). ^13^ Further, the relatively low cost and rapidity of conducting bibliometric analyses allow for replicable methods. ^13^^,^^14^ Use of bibliometric techniques across different scientific fields is becoming increasingly popular. ^15^^,^^16^

A scoping review, which involves mapping the current literature and identifying gaps in research, ^17^ would be appropriate to summarize literature on CAIM bibliometric analyses. To the best of our knowledge, no systematic or scoping reviews have been conducted on bibliometric analyses of CAIM therapies. A preliminary search of the Cochrane Database of Systematic Reviews and the Scopus database revealed no existing systematic or scoping reviews on the topic. Synthesizing bibliometric analyses on CAIM will provide insight into trends, such as the types of CAIM literature typically analysed from a bibliometric lens, statistical techniques that bibliometric analyses on CAIM utilize, and more broadly, where the field of CAIM is headed. Considering the lack of standardized procedures for bibliometric analyses, this review will also improve understanding of how bibliometric analyses are currently conducted on this topic. Thus, the purpose of this review will be to understand the characteristics of bibliometric analyses of CAIM research literature, which can inform future work within the field.

## Protocol

### Methods

*Approach and eligibility criteria*

The present scoping review’s research question is: “What are the characteristics of bibliometric analyses of the CAIM literature?”. The proposed scoping review will be conducted in accordance with the Joanna Briggs Institute (JBI) methodology for scoping reviews, which recommends quantitative and descriptive analyses of main findings. ^17^ This protocol’s associated files (Appendix A – Search Strategies, and the PRISMA ScR Checklist) has been made openly available on Open Science Framework (see *Extended data* and *Reporting guidelines* ^33^).

The search strategy will aim to locate studies published in journals only (excluding grey literature), with no date restrictions (i.e., from inception to date of search execution). The only eligible study design will be bibliometric analyses (encompassing terms for “bibliometric analysis”, “scientometric analysis”, and “citation analysis”), or articles that include both a bibliometric analysis and another study design (e.g., bibliometric analysis and systematic review). All included bibliometric analyses will be focused on one or more CAIM therapies, as defined by a recently published operational definition of CAIM. ^18^ This operational definition was created using a systematic search of four peer-reviewed or other quality-assessed resource types: 1) peer-reviewed articles from seven major bibliographic databases, 2) “Aims and Scope” webpages of peer-reviewed CAIM journals, 3) entries containing CAIM therapies in highly accessed online encyclopedias, and 4) highly ranked websites resulting from Health On the Net Code of Conduct (HONcode) searches. ^18^ To date, this operational definition includes the greatest number of evidence sources and is the only one that captures the concept of “integrative medicine”, alongside “complementary medicine” and “alternative medicine”. ^18^

Grey literature sources will be excluded as bibliometric analysis studies are unlikely to be found outside of traditional academic publishing channels. Conference abstracts and study protocols will also be excluded as they likely will not contain adequate information required to describe the characteristics of bibliometric analyses on CAIM literature. Finally, all non-English publications will be excluded, due to language constraints of the authors.

*Search strategy*

The following electronic databases will be searched: MEDLINE, EMBASE, PsycINFO, and AMED (accessed via the OVID research platform), as well as CINAHL (accessed via EBSCOhost), Scopus, and Web of Science. The search strategy will include a comprehensive search string of CAIM terms ^19^ encompassing 604 distinct therapies described previously in an operational definition of CAIM. ^18^ This search string of CAIM terms was created for OVID (e.g., MEDLINE, EMBASE, PsycINFO, AMED) and EBSCO platform (e.g., CINAHL) databases, as well as Scopus and Web of Science databases. ^19^ Relevant scientific names and/or synonyms were added as a term (i.e., keyword, phrase), alongside relevant boolean operators. The comprehensive search string of CAIM ^19^ will be combined with search terms for bibliometric analyses (e.g., bibliometric analysis, statistical bibliography, citation analysis). The search strategy, including all identified keywords and equivalent index terms, will be adapted for each included database. All search strategies that will be run are provided in *Extended data,* ^33^ informed by PRISMA-S guidelines for reporting literature searches. ^20^^,^^33^

*Study and source of evidence selection*

Following the search, all identified citations will be collated and exported into Covidence, and duplicates will be removed. All titles/abstracts, followed by full texts, will be screened by reviewers independently and in duplicate. First, pilot title/abstract screening of a sample of twenty articles will be conducted by AQS and HL. A meeting will be held between AQS, HL, and JYN to discuss any challenges and resolve discrepancies. Following the pilot test, all titles/abstracts will be screened for inclusion by AQS and HL. Then, pilot full-text screening will be conducted, in which AQS and HL will screen ten full texts. A meeting will be held between AQS, HL, and JYN to discuss challenges and resolve discrepancies. After this pilot step, full-text screening will be completed by AQS and HL. Reasons for exclusion of full texts will be recorded. Any disagreements that arise between reviewers throughout the selection process will be resolved on a weekly basis through discussion with HL, AQS, and JYN, if disagreements still cannot be resolved. The results of the search strategy and the study screening process will be presented in a PRISMA-ScR flow diagram in the final scoping review. ^21^

*Data extraction*

Data extraction will be conducted using Excel software. The data extraction form that will be used in this scoping review is informed by Donthu *et al*., ^13^ which provides an overview of how to conduct a bibliometric analysis. The extraction form will be developed in two stages. In stage one, AQS and HL will select ten articles at random that met the inclusion criteria from a preliminary search of CAIM and bibliometric analysis search terms on Scopus. AQS and HL will independently extract information from five articles each and meet to discuss discrepancies. A meeting will be held among AQS, HL, and JYN, to discuss changes needed to improve the form. In stage two, AQS and HL will identify the ten most highly cited articles that met inclusion criteria after running a search of CAIM literature and bibliometric analyses terms on Scopus, before independently extracting information from five articles each using the latest version of the data extraction form. Another meeting will be held between AQS and HL, and then with JYN, to approve the latest version of the extraction form.

The following information will be extracted from eligible bibliometric analyses: title, author, year, country, aim of the study, secondary study design (if applicable), the type of CAIM(s), the health condition or population targeted, main findings, conclusions, and limitations. Also, the bibliometric information described will be summarized, including the databases searched, type of bibliometric methodology (i.e., performance analysis [such as citation-metrics or publication metrics] versus science mapping [such as co-word analysis, co-authorship analysis, bibliographic coupling, or enrichment techniques]), the number of studies included in the analysis, the number of metrics used, how information was reported (e.g., narrative summary, figures, tables, visualization software used), and how all variable measures align with the Donthu *et al*. ^13^ guideline for conducting bibliometric analyses.

To ensure consistency and quality of the extraction, an initial pilot test of data extraction will be conducted by all participating independent reviewers, assessing five articles. All reviewers will then meet with JYN, HL, and AQS to resolve discrepancies and disagreements. Based on the initial pilot testing, revisions to the data extraction form can be proposed and implemented. Upon completion of the pilot extraction step, reviewers will be divided into two teams led by HL and AQS. Teams will be further divided into pairs of two reviewers for duplicate data extraction of the same set of bibliometric full texts. These duplicate extractions will be reviewed by AQS and HL to ensure consistency. Weekly meetings will be held between reviewers and HL and AQS to standardize the data extraction process and resolve any issues identified. Any conflicts that cannot be resolved will be discussed with JYN.

We seek to conduct a scoping review. As we are going to use the JBI methodology for scoping reviews, we have elected not to conduct a risk of bias assessment.

### Data analysis and presentation

The data will be summarized descriptively (e.g., frequencies of the number of studies, country, types of CAIM, outcomes reported in the bibliometric analyses) with full results presented in tabular format. Frequency of CAIM bibliometric analysis publications over time will be presented in graphical format. An additional figure will be created highlighting the types of bibliometric information each study reported.

*Dissemination*

The findings of this review will be disseminated in scientific journals.

### Study status

The literature search is ongoing.

## Discussion

We anticipate this project will advance the understanding of topics and trends in CAIM research, including what types of CAIMs are most commonly explored and what health conditions these CAIMs target. Accordingly, it can help CAIM researchers locate bibliometric sources to inform future research directions or identify gaps that warrant further investigation. It is anticipated that this review will also provide unique insights on how bibliometric analyses of CAIM literature are conducted, including the type of methodology used (such as performance analysis metrics or science mapping techniques), the number and types of outcomes reported, and how bibliometric information is presented (e.g., narratively, graphically).

As the number of scientific publications has been growing exponentially in the last fifty years, bibliometrics has become useful to quantitatively analyse publications on a particular topic. ^22^ Generally, there is large variability in how bibliometric analyses are conducted, as there is no authoritative guideline on bibliometric methodology. ^13^^,^^16^ Findings from the completed review can help identify whether there are any inconsistencies in the way that bibliometric analyses specifically on CAIM literature are conducted. This may be useful to inform future, standardized reporting guidelines for bibliometric analyses, generally, or with a potential focus on CAIM topics. Further, it may be expected that performance analysis metrics used to measure research constituents’ scientific impact (e.g., h-index, g-index, total citations) differ between bibliometric analyses. ^23^ Given the varying capability of different performance analysis metrics in capturing scientific impact of a given research constituent, ^24^ this review could reveal the extent to which bibliometric analyses of CAIM literature are effectively measuring scientific impact. Comparisons of different research constituents’ scientific impact are further complicated by how average values of bibliometric indicators often differ between disciplines (e.g., molecular biology, nursing). ^22^ This is particularly pertinent to CAIM literature, given the interdisciplinary applications of many CAIM therapies. ^25^^–^^27^

Bibliometric methodology can be influenced by database changes such as the indexing of new journals or articles. ^28^ Due to limitations of visualization softwares like VOSviewer, often only either Scopus or Web of Science databases can be searched, which cover most but not all databases. ^29^ If the findings of this scoping review reveal similar limitations of bibliometric analyses conducted on CAIM literature, these limitations could potentially drive investigation into techniques that will improve analysis of bibliometric results. Additionally, there are no known critical appraisal tools for bibliometric analyses. Concerns have been expressed over a lack of knowledge of good practices when conducting bibliometric analyses. ^30^ While this is outside the scope of this present review, future research may investigate assessment tools to evaluate the quality of published bibliometric analyses. The identification of a group of bibliometric analyses in this review may potentially serve as a test set for the development and investigation of quality indicators.

### Strengths and limitations

Strengths of this study will include adherence to the JBI methodology for scoping reviews ^17^ and use of a comprehensive systematic search strategy ^19^ across several databases to identify eligible articles. Another strength is that screening and data extraction will be conducted in duplicate, significantly reducing bias. There are some limitations expected in this review. By only including studies written in English, we could be missing important international work. For example, Chinese databases may contain a higher volume of CAIM articles but are unable to be searched in this study. This is especially relevant as some forms of CAIM may be practiced more frequently in non-English speaking regions of the world, such as traditional Chinese medicine in China. ^31^^,^^32^ Additionally, reported findings are expected to be descriptive in nature, such as the frequencies of the types of CAIMs examined in bibliometric studies or the frequencies of studies that engaged in science mapping versus performance analysis techniques. This makes it difficult to extrapolate themes or correlates, like which CAIMs are effective, or which bibliometric techniques are preferable.

## Data Availability

No underlying data are associated with this article. Open Science Framework: Characteristics of Bibliometric Analyses of Complementary, Alternative, and Integrative Medicine Literature: A Scoping Review. https://doi.org/10.17605/OSF.IO/JSQWY. ^33^ This project contains the following extended data:
- Appendix A - Search Strategies_Jan0923.docx (search strategies for MEDLINE, EMBASE, PsycINFO, AMED, CINAHL, Scopus and Web of Science databases). Open Science Framework: PRISMA-ScR checklist for ‘Characteristics of bibliometric analyses of the complementary, alternative, and integrative medicine literature: A scoping review protocol.’ https://doi.org/10.17605/OSF.IO/JSQWY. ^33^ Data are available under the terms of the Creative Commons Attribution 4.0 International license (CC-BY 4.0).
