# Supplementary material for: Characteristics of bibliometric analyses of the complementary, alternative, and integrative medicine literature: A scoping review protocol
Source: F1000Res. 2023 Oct 18;12:164. Originally published 2023 Feb 13. [Version 2] doi: 10.12688/f1000research.130326.2 (PMC10905119; doi:10.12688/f1000research.130326.2)
Supplement: Peer review history

# Reviewer report: Reviewer response for version 2

**DOI:** 10.5256/f1000research.157140.r237990

**Published:** 2024-02-13

**Authors:** Zhengwei Huang

**Affiliations:** 1 Jinan University, Guangdong, China

Thank you very much for inviting me to review this Study Protocol. I noticed that this paper entitled “Characteristics of bibliometric analyses of the complementary, alternative, and integrative medicine literature: A scoping review protocol” was a revised version. It proposed a detailed research protocol for the bibliometric scoping review of CAIM literatures. The background was clearly demonstrated, and the methodology was acceptable. The other sections like discussions were okay. Based on my personal research experience on bibliometric analysis, I supposed that the protocol was probably feasible. Most importantly, the authors had well responded to the previous comments, and made proper revisions. Therefore, basically it can be Approved for indexing. An expectation is that, as the authors stated in the Dissemination Section, “the findings of this review will be disseminated in scientific journals”, we can see the full report as soon as possible.

Is the study design appropriate for the research question?

Yes

Is the rationale for, and objectives of, the study clearly described?

Yes

Are sufficient details of the methods provided to allow replication by others?

Yes

Are the datasets clearly presented in a useable and accessible format?

Yes

Reviewer Expertise:

Bibliometrics; Nanomedicine; Drug delivery; Ferroptosis

I confirm that I have read this submission and believe that I have an appropriate level of expertise to confirm that it is of an acceptable scientific standard.

# Reviewer report: Reviewer response for version 2

**DOI:** 10.5256/f1000research.157140.r216587

**Published:** 2023-10-19

**Authors:** Robert Trager

**Affiliations:** 1 Connor Whole Health, University Hospitals Cleveland Medical Center, Cleveland, OH, USA

The authors made adequate improvements to their manuscript and provided a concise summary of the changes. I have no further comments. I congratulate them and look forward to seeing the review itself.

Is the study design appropriate for the research question?

Yes

Is the rationale for, and objectives of, the study clearly described?

Yes

Are sufficient details of the methods provided to allow replication by others?

Yes

Are the datasets clearly presented in a useable and accessible format?

Not applicable

Reviewer Expertise:

chiropractic, CAIM

I confirm that I have read this submission and believe that I have an appropriate level of expertise to confirm that it is of an acceptable scientific standard.

# Reviewer report: Reviewer response for version 1

**DOI:** 10.5256/f1000research.143074.r163736

**Published:** 2023-08-14

**Authors:** Robert Trager

**Affiliations:** 1 Connor Whole Health, University Hospitals Cleveland Medical Center, Cleveland, OH, USA

**Overview**

 I congratulate the authors on an extremely well-written, thorough, and clear protocol for a scoping review of bibliometric studies on CAIM. They adequately justify the rationale for the study and describe how their findings may impact the CAIM literature. The methodology is concise yet descriptive. I also appreciated that open-access files were included on OSF, along with an impressive search strategy. I have no major comments regarding the protocol, and only a couple of minor comments.

**Minor comments**

1. The authors note that their study will help identify “ *what health conditions these CAIMs target,*” and in the abstract state “ *health condition targeted.*” While this is certainly OK, I believe that the language could be slightly altered in these sections of text. I expect that the authors may encounter bibliometric studies of CAIM which describe not only the treatment of certain conditions, but also allude to a diagnostic, clinical reasoning, case management process, or the use of CAIM preventative purposes among individuals who are already healthy (e.g., “wellness”). For example, in our bibliometric study of chiropractic case reports, we found an increasing trend in studies describing the diagnosis of vascular disorders. While I am less familiar with other fields, I imagine a similar phenomenon may be noted for CAIM-related professions wherein providers have a broad scope of practice, requisite on diagnosis, such as osteopathy or physical therapy. I think the protocol could be therefore altered slightly to change “target(ed)” to “manage(ed)” or some other language that is broader to reflect more than just treatment, but rather an overall management of the patient.
2. The authors describe CAIM as “therapies” throughout the manuscript. I think they have the liberty to describe it this way, yet I would caution them that in some instances the CAIM therapy is distinct from the branch of providers that often use that therapy. For example, in my field of chiropractic, chiropractors often use spinal manipulation, yet chiropractors also perform diagnosis and referral in their management of health conditions and sometimes omit spinal manipulation altogether. One might also consider that a single CAIM therapy could be provided by several types of practitioners. To continue with the above example, osteopaths and physical therapists also use spinal manipulation. The authors could have an a priori method for handling how they categorize CAIM therapies versus practitioner types. However, given that this may be confusing and/or unnecessary to establish before seeing the results, the authors could also describe the categorization of CAIM therapies versus provider types as an iterative process, subject to change if there is overlap between the two.

**Comments for clarity**

1. Data extraction – The phrase “We seek to conduct a scoping review” is redundant and could be deleted.

This is totally optional as it’s not directly related to the manuscript but may be helpful for other readers – on OSF I could not preview the two Appendix files and had to download them to be able to see them. Consider uploading the files as a PDF in OSF so they can be viewed within an internet browser. However, I recommend also keeping the original Word documents on OSF.

Is the study design appropriate for the research question?

Yes

Is the rationale for, and objectives of, the study clearly described?

Yes

Are sufficient details of the methods provided to allow replication by others?

Yes

Are the datasets clearly presented in a useable and accessible format?

Not applicable

Reviewer Expertise:

chiropractic, CAIM

I confirm that I have read this submission and believe that I have an appropriate level of expertise to confirm that it is of an acceptable scientific standard, however I have significant reservations, as outlined above.

# Response

**Published:** 2023-10-06

**Authors:** Jeremy Ng

**Affiliations:** University of Ottawa, Canada

We kindly thank the reviewer for their feedback. 

 Regarding *minor comment 1*, we agree that the language can be changed from “target(ed)” to “manage(d)” to account for the fact that not all bibliometric analyses may specifically discuss treatment, but rather provide a broad overview of the field in regards to diagnostics, clinical reasoning, or condition management. We have implemented these changes to the wording in the Abstract, 2nd paragraph of Data Extraction, and 1st paragraph of Discussion. Additionally, we will also be extracting main findings, conclusions and limitations of each bibliometric analyses, which is another way that we are able to capture some of the interesting findings the reviewer mentioned that goes beyond the “treatment” of conditions. 

 Regarding *minor comment 2 *and the terminology of CAIM as a “therapy”, to define the types of complementary, alternative, and integrative therapies (CAIM) eligible for inclusion in our review, we are using a Cochrane Complementary Medicine-adopted operationalized list of CAIMs ^1^, which are described as “CAIM therapies”. For consistency, we are using the same terminology. However, the operationalized list is inclusive of many provider types/branches, including chiropractic and osteopathic fields. The data extraction of “CAIM therapies” will be reported in accordance with terminology used by the operationalized list.

 We also find the reviewer’s point interesting and acknowledge that different CAIM practices or techniques (e.g., spinal manipulation) may be employed by different types of practitioners. However, we anticipate that not all bibliometric analyses included in our review would include the same types of metrics. Accordingly, not all bibliometric analyses may report the types of providers that are providing these CAIM therapies in the literature. In our data extraction form, we will report whether a paper has included the “types of providers” as a metric in their bibliometric analysis. However, we will unfortunately be unable to extract extensive details on which providers would be providing these therapies. This is for the sake of consistency across all the studies, and is in line with JBI scoping review methods which aims to map and provide a broad overview of the literature. To your point, it would be difficult to devise methods for categorizing CAIM therapies versus provider types as described. 

 Regarding the *comments for clarity*, we agree that the phrase “We seek to conduct a scoping review” is redundant and have removed it from the protocol. Additionally, we will upload PDF versions of the two Appendix files, alongside the Microsoft Word document files, in Open Science Framework. 

**References**

1. Ng JY, Dhawan T, Dogadova E, Taghi-Zada Z, Vacca A, Wieland LS, Moher D. Operational definition of complementary, alternative, and integrative medicine derived from a systematic search. *BMC. Complement. Altern. Med*. 2022;22(1):104. doi: 10.1186/s12906-022-03556-7

# Reviewer report: Reviewer response for version 1

**DOI:** 10.5256/f1000research.143074.r181379

**Published:** 2023-08-14

**Authors:** Jenny-Ann Brodin Danell

**Affiliations:** 1 Umeå University, Umeå, Sweden

Thanks for the protocol! The introduction is relevant and ok, both regarding definition of central concepts, relevance of analyzing CAIM literature, and what/how bibliometric methods can be helpful for that.

 However, I would prefer a more elaborated motivation of the study. Why is it relevant to analyze the characteristics of bibliometric studies of CAIM? Are there reasons to expect them to be different from other medical/health sub-fields? Are there particular pitfalls? And isn’t it more or less necessary to compare these studies with other areas to be able to say something relevant about the patterns (at least indirectly? I have not checked the details, but there seem to be a few similar scoping reviews on other medical sub-fields.)

 The search procedure/extraction seems appropriate, as well as the choice of databases. Characteristics/information that will be extracted is also relevant in relation to the scope of the study.

 A minor detail; the formulations about lack of standardized procedures in bibliometric analysis just seem blunt. Bibliometrics is, as you know, a diverse field of research methods and there are never ending discussions about limitations, potential developments (etc.), but there are certainly standardized procedures.

 Looking forward to the results!

Is the study design appropriate for the research question?

Yes

Is the rationale for, and objectives of, the study clearly described?

Partly

Are sufficient details of the methods provided to allow replication by others?

Yes

Are the datasets clearly presented in a useable and accessible format?

Not applicable

Reviewer Expertise:

Sociology, bibliometrics, medical sociology

I confirm that I have read this submission and believe that I have an appropriate level of expertise to confirm that it is of an acceptable scientific standard, however I have significant reservations, as outlined above.

# Response

**Published:** 2023-10-06

**Authors:** Jeremy Ng

**Affiliations:** University of Ottawa, Canada

We kindly thank the reviewer for their feedback.

 Regarding our motivations for conducting this study, we are particularly interested in mapping bibliometric analysis studies of CAIM, as, to our knowledge, no systematic or scoping reviews have been completed on this topic. The prevalence of CAIM use is increasing worldwide, and the body of CAIM literature has grown immensely in the past two decades, as we mentioned in our introduction section. Bibliometric analyses of CAIM would capture interesting trends in the field such as the publications over time of different CAIM therapies, or the countries that contribute most to particular research topics. This may be useful to CAIM researchers to identify gaps in the CAIM field and where research is headed. We have stated these points in the last paragraph in the introduction section. 

 Further, analyzing the characteristics of bibliometric analyses of CAIM literature can allow us to identify commonly and less commonly used bibliometric analysis metrics across studies. This could be of interest to inform the development of reporting guidelines for bibliometric analyses. As stated in the introduction section, while there are guidelines for how to conduct bibliometric analyses, there is no consensus in the literature on what should be included. We anticipate that articles may have diverse or unique bibliometric variables, or may even range in the number of variables used. As stated above, this could be useful to improve our understanding of how bibliometric analyses are conducted, and can help to inform future reporting guidelines on this methodology. 

 Regarding your suggestion on comparing characteristics of CAIM bibliometric analyses to other fields, we agree this would be interesting. We have not been able to locate systematic or scoping reviews that report the characteristics of bibliometric analyses (e.g., performance analysis or science mapping techniques used, main findings, health conditions managed) of CAIM literature or of other medical/health sub-fields. Accordingly, it is difficult to ascertain how our findings would compare to other medical/health sub-fields. We would be interested if the reviewer is able to share any identified review articles exploring the characteristics of bibliometric analyses in other medical fields.  

 Regarding the standardized procedures of bibliometrics, we acknowledge that bibliometric analyses can be conducted in diverse ways, and there are some guidelines on the types of metrics that can be used (e.g., Linnenleucke et al., 2019 ^1^; Donthu et al., 2021 ^2^). For example, we plan to use the paper by Donthu et al. ^2^ to inform our scoping review. However, there is a lack of consensus in the literature on what baseline information should be required as part of a bibliometric analysis, unlike other research designs (e.g., PRISMA guidelines and JBI methods have been widely adopted for reporting and conduct of systematic reviews). We have rephrased the sentence in the 5th paragraph of the Introduction section to reflect that standardized procedures exist, but that there is no universal standard or consensus in the literature on what a bibliometric analysis should at minimum entail.

**References**

1. Linnenluecke MK, Marrone M, Singh AK. Conducting systematic literature reviews and bibliometric analyses. *Aust. J. Manag.* 2020;45(2):175-194. doi: 10.1177/0312896219877678
2. Donthu N, Kumar S, Mukherjee D, Pandey N, Lim WM. How to conduct a bibliometric analysis: An overview and guidelines. *J. Bus. Res*. 2021;133:285-296. doi: 10.1016/j.jbusres.2021.04.070
